# Anaesthetic considerations in Shrinking Man syndrome: two case reports

**DOI:** 10.1186/s12871-023-01978-5

**Published:** 2023-01-12

**Authors:** Yan Xu, Ying Hong, Xin Liu, Li Zhou, Chunling Jiang

**Affiliations:** 1 Department of Anesthesiology, West China Hospital, Sichuan University & The Research Units of West China (2018RU012), Chinese Academy of Medical Sciences, Chengdu, 610041 China; 2 Department of Anesthesiology and Translational Neuroscience Center, West China Hospital, Sichuan University, 610041 Chengdu, China

**Keywords:** Shrinking Man Syndrome, Difficult airway, Mitral stenosis, Hypotension, Case report, Anaesthetic consideration

## Abstract

**Background:**

Shrinking Man syndrome (SMS) is a rare but often serious complication of dialysis-dependent end-stage renal disease, characterized by significant loss of height, bone pain, bone deformity, and skin itching. Patients with SMS always have abnormal facial changes and cardiovascular system damage (manifested by hypertension, hypotension, cardiovascular calcification, and valvular heart disease), which pose a great challenge to anaesthesiologists. The purpose of this report is to describe our anaesthetic experience regarding two patients with SMS combined with alterations of the airway and cardiovascular system.

**Case presentation:**

We describe two cases of SMS treated at West China Hospital, a tertiary care centre in Chengdu, China. All cases met the diagnostic criteria, which comprised 1) dialysis-dependent end-stage renal disease, 2) loss of height, and 3) bone pain and bone deformity. One patient had an anticipated difficult airway and moderate-to-severe mitral stenosis. The other patient presented with significant hypotension. Anaesthetic considerations included awake fibreoptic bronchoscopy-assisted tracheal intubation, real-time transoesophageal echocardiogram monitoring and individualized blood pressure management strategies.

**Conclusion:**

This case series highlights the importance of adequate preoperative assessment and preparation, as well as individualized anaesthetic management, in patients with SMS.

## Background

Shrinking Man syndrome (SMS) is a rare but serious complication of dialysis-dependent end-stage renal disease (ESRD), characterized by significant loss of height, bone pain, bone deformity, and skin itching. Inadequate treatment for secondary hyperparathyroidism (SHPT) is predominantly responsible for SMS. Parathyroidectomy is an effective treatment for SHPT. In addition to the damage caused by prolonged dialysis (such as coagulation disorders, hypoproteinaemia, renal anaemia, hypertension, etc.), some patients with SMS also develop abnormal facial changes, cardiovascular calcification, and soft tissue calcification. These pathophysiological changes, notably those involving the airway and cardiovascular system, pose enormous challenges to anaesthesiologists. Consideration of the relevant anaesthetic management is currently extremely limited. Herein, we report our anaesthetic experience regarding two SMS patients who had combined changes in the airway and the cardiovascular system. Written informed consent was obtained from all patients.

## Case presentation

### Case one

**Fig. 1:**
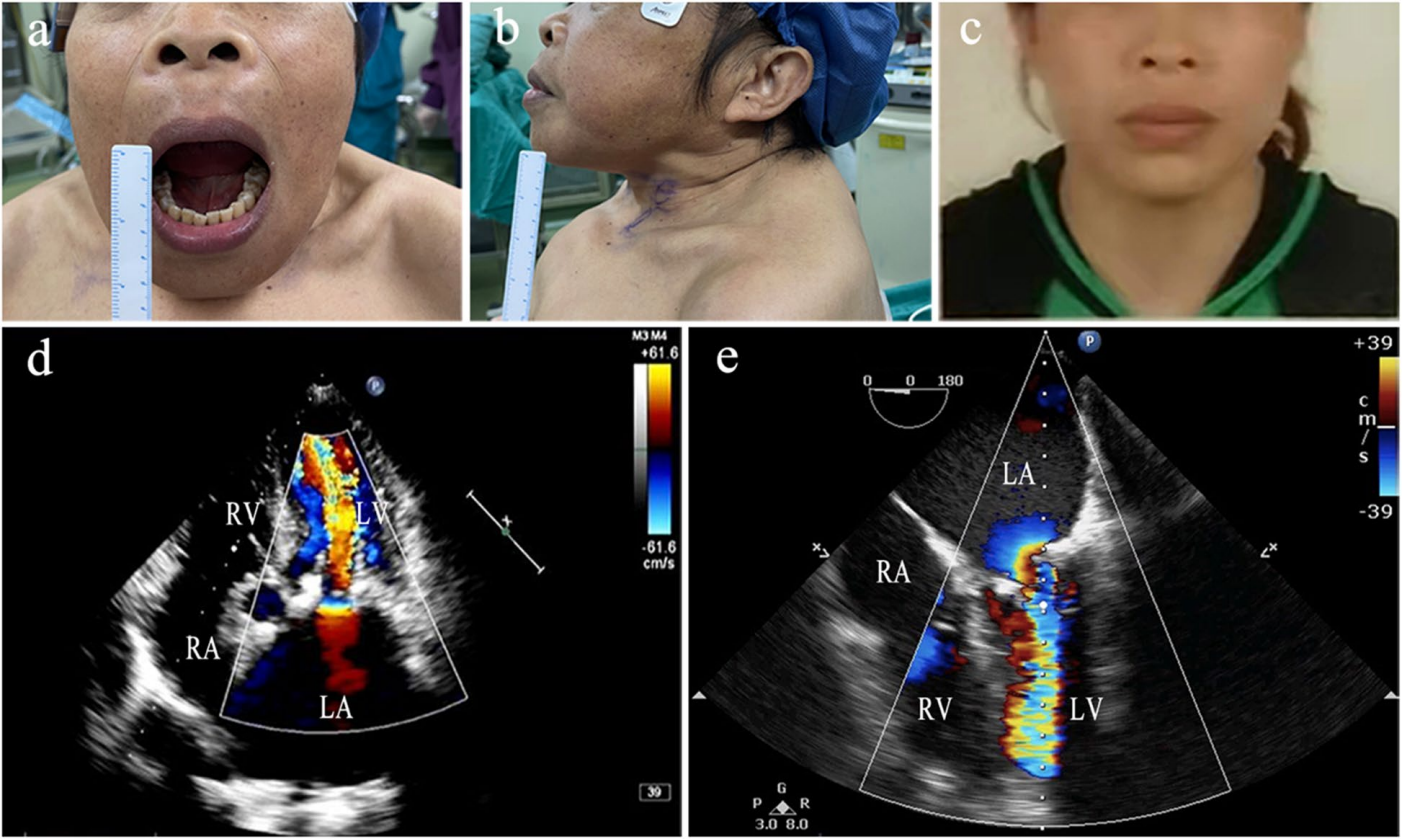
(**a**, **b**) Current abnormal human face appearances (deformity of the maxilla and mandible, flattening of nasal cartilage); (**c**) Previously taken normal facial photograph; (**d**) Transthoracic echocardiography findings before operation; (**e**) Transoesophageal echocardiography findings after induction of anaesthesia

A 49-year-old American Society of Anaesthesiologists (ASA) Physical Status III female patient (weight, 35 kg; height, 140 cm) presented with height loss and facial changes. She had a history of dialysis-dependent ESRD for 11 years, hypertension for over 11 years, and SHPT for over 8 years. One year ago, she noticed that her face had changed and that some (10 cm) of her height had been lost (Fig. 1a-c). She also felt mild chest discomfort during ordinary activity. Pertinent laboratory examination included a serum parathyroid hormone (PTH) level of 518 pg/mL, serum creatinine of 550.0 μmol/L, serum calcium of 2.56 mmol/L, and B-type natriuretic peptide (BNP) of 11,097 ng/L. After repeated treatment, the symptoms did not improve. The patient was then scheduled for parathyroidectomy. The admission diagnosis was ESRD, hypertension, SHPT, SMS, mitral stenosis (moderate to severe), coronary artery diseases, New York Heart Association (NYHA) Classification III. Preoperative ECG showed sinus rhythm. Preoperative transthoracic echocardiography (TTE) revealed obvious calcification of the mitral annulus with moderate-to-severe stenosis (estimated mitral orifice size, 1.0 cm^2^) and moderate pulmonary hypertension (pulmonary artery systolic pressure of 57 mm Hg) (Fig. 1d). The coronary angiography showed 40% obstruction in the proximal part of the left anterior descending artery. The preoperative airway assessment included Mallampati class IV, a 5 cm sternomental distance, a 2.5 cm interincisor distance, and restricted neck mobility. An airway management plan for anticipated difficult airway was prepared, including topical anaesthesia for awake tracheal intubation, sedative and analgesic drugs, and fibreoptic bronchoscopy guidance.

The patient underwent routine monitoring, and baseline preinduction vital signs included heart rate (HR) 70 beats per minute, arterial blood pressure (ABP) 165/90 mm Hg, and oxygen saturation 98% on room air. After adequate preoxygenation, she was given 2 mg of midazolam, 1 mg of penehyclidine hydrochloride and 5 μg sufentanil intravenously. Additionally, dexmedetomidine was administered at a dose of 0.4 μg.kg^−1^.h^−1^ via an intravenous infusion pump, which was ceased 45 min before the end of the operation. Then, cricothyroid membrane puncture was performed under ultrasound guidance by using a 23G needle, and 3 ml of 2% lidocaine was injected following verification of intratracheal placement by performing air aspiration. After injection, the patient was asked to cough to transport the local anaesthetic from the tracheal injection site to the subglottic mucosa. Subsequently, 10 ml 2% lidocaine jelly was gargled for 10 min. A laryngeal tube was used to test the sensation in the back of the oropharynx. If there was a pharyngeal reflex, 2 ml of 2% lidocaine was sprayed. After sufficient topical anaesthesia and sedation, a skilled anaesthesiologist inserted an EMG endotracheal tube (ID 6.0 mm) through the mouth under the guidance of fibreoptic bronchoscopy, without obvious haemodynamic fluctuations. Thereafter, intravenous anaesthetic induction was safely accomplished after the endotracheal tube was secured. Transoesophageal echocardiography (TEE) was performed to monitor cardiac function and targeted circulatory management in real time (Fig. 1e) after deepening anaesthesia. The operation went smoothly. During the operation, the patient’s vital signs were stable. After the recovery of consciousness and return of spontaneous respiration, the tracheal tube was removed, and the patient was safely transferred to the intensive care unit. The patient was discharged 1 week later without any complications. There were also no complications at the 3-month follow-up.

### Case two

**Fig. 2:**
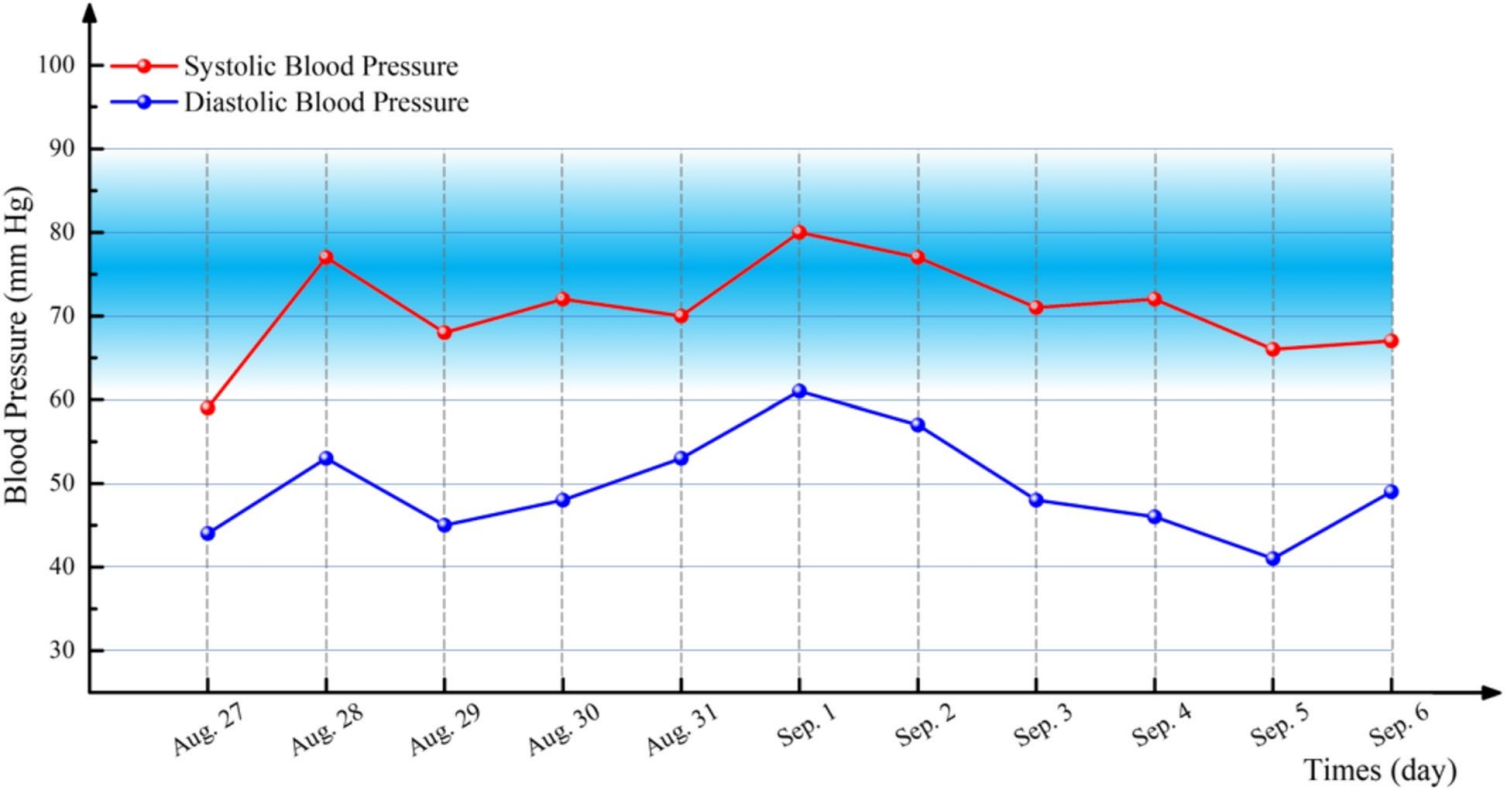
Fluctuation in blood pressure levels before surgery

A 53-year-old, 48 kg, 141 cm, and ASA III level female had a history of 10 years of dialysis-dependent ESRD and 3 years of bone pain, height loss of 10 cm and significant hypotension. Her blood pressure ranged from 50–70 mm Hg systolic and 35–50 mm Hg diastolic, without dizziness, chest pain, or other symptoms. The laboratory examination suggested that the level of PTH was increased, and repeated treatment was ineffective. Thus, she was scheduled for parathyroidectomy in our institution. The admission diagnosis was ESRD, SHPT, SMS, hypotension, and NYHA Classification II. Preoperative laboratory examination included a serum PTH level of 339 pg/mL, serum creatinine of 266.0 μmol/L, serum calcium of 2.48 mmol/L, and BNP 3180 ng/L. ECG revealed sinus tachycardia (127 bpm). Before the operation, her blood pressure fluctuated between 59–80 mm Hg systolic and 41–61 mm Hg diastolic at rest (Fig. 2), without dizziness, chest pain or other symptoms. Preoperative airway evaluation included a Mallampati class II, a 4 cm interincisor distance, and normal neck mobility.

The patient underwent routine monitoring after entering the operating room. The preinduction vital signs included the following: HR: 82 beats per minute, ABP: 78/60 mm Hg, and SpO_2_: 98% on room air. After preoxygenation was achieved with 100% O_2_, 16 mg etomidate, 15 μg sufentanil and 10 mg cisatracurium were given during anaesthesia induction. Immediately following induction, systolic BP dropped to 47 mm Hg, it was managed promptly with 0.2 mg metaraminol injection. Following hemodynamic stabilization, an EMG endotracheal tube (ID 6.0 mm) was successfully intubated under video laryngoscopy exposure after complete neuromuscular blockade. Intraoperatively, the HR, SpO_2_, ECG, End-tidal carbon dioxide, ABP and bispectral index (BIS) were monitored. Throughout the remaining intraoperative period, anaesthesia was maintained with remifentanil 0.1–0.2 μg.kg^−1^.min^−1^ and desflurane in a mixture of air (40%) and O_2_ (60%) to maintain the BIS within 40 to 60. Systolic blood pressure was targeted to remain within ± 10% of the baseline level (blood pressure that she maintained at home). The operation went smoothly. No other adverse event was observed. Fluid intake was 400 ml Ringer’s lactate and blood loss was 10 ml. The surgery lasted 1.3 h. Following extubation, the patient was transferred to the postanaesthetic care unit. She was discharged on the 7th day after the operation. At the 3-month follow-up, the patient's blood pressure still fluctuated between 75–90/50–70 mm Hg, but no related complications occurred.

## Discussion and conclusion

In 1980, Horensten et al. [1] first reported a special case of a patient who had a consequent 28-cm loss in height after haemodialysis for 9 years, which was later labelled as SMS. In addition to the pathophysiological features inherent to dialysis, patients with SMS also exhibit various changes in the skeletal system as the disease progresses. In some severe cases, abnormal facial changes (manifested by difficult airway) and cardiovascular system damage (manifested by hypertension, hypotension, cardiovascular calcification, or valvular heart disease) may occur [2]. The Kidney Disease: Improving Global Outcomes (KDIGO) guidelines have proposed [3] that parathyroidectomy is the preferred option to avoid further disease progression and improve the patient's well-being if the patient progresses to the refractory stage of SHPT. However, abnormal alterations in the face and cardiovascular system undoubtedly pose a great challenge to anaesthesiologists.

For the first patient described above, the most significant pathophysiological alternative was the aberrant facial changes, characterized by an overall enlargement of the maxilla forwards and downwards, resulting in a "hippopotamus"-like change in the patient's entire face. This unusual clinical syndrome caused by SHPT was also named Sagliker syndrome by Sagliker et al. in 2004 [4]. The patient belonged to Mallampati class IV and demonstrated limited neck mobility and restricted mouth opening (only approximately 2.5 cm), making it difficult to perform laryngoscopy. Thus, we opted for awake tracheal intubation guided by fibreoptic bronchoscopy via the mouth. Awake tracheal intubation has a high success rate and a low-risk profile and has been cited as the gold standard in airway management for an anticipated difficult airway [5], and fibreoptic bronchoscopy is by far the most commonly recommended guiding tool [6]. For such patients, the indication for extubation should also be strictly controlled.

Apart from the difficult airway, this patient also presented moderate-to-severe mitral stenosis, moderate pulmonary hypertension, and coronary artery diseases. Its pathogenesis appears to be due to abnormal calcium-phosphorus homeostasis in the setting of SHPT. The main pathophysiological variation of mitral stenosis is that the stenotic mitral valve prevents the left atrium from emptying properly. Finally, pulmonary hypertension and right heart failure have occurred in severe cases [7]. The occurrence of valve disease, pulmonary hypertension, and coronary artery diseases dramatically enhances the risk of anaesthesia. Our objectives in anaesthetic management are to provide adequate analgesia, reduce the release of endogenous catecholamines, and avoid a rapid ventricular rate to maintain the balance between oxygen supply and demand. It is pertinent to add that we should also avoid hypoxia and carbon dioxide accumulation, which aggravate pulmonary hypertension. Additionally, monitoring is also an indispensable step in helping patients to successfully pass through the perioperative period. Therefore, we used TEE to monitor the cardiac structure and function, preload status and other conditions of patients in real time. With the continuous development of ultrasound technology, the application of TEE has been extended to many noncardiac operations [8]. Notably, this is the first report on the use of TEE in patients with SMS and valve heart disease.

SHPT has long been associated with arterial hypertension, which may be related to renal insufficiency [9]. Unexpectedly, our second patient presented with significantly persistent low blood pressure without dizziness, chest pain, ST-segment abnormalities on the ECG or significant ventricular wall motion abnormalities when assessed by a TTE. The underlying mechanisms in this case are poorly understood. It is speculated that the persistent hypotension of this patient may be related to elevated PTH, which has a direct vasodilatory effect, or indirectly increased prostaglandin secretion [10]. Individualized blood pressure management strategies in perioperative care (systolic blood pressure targeted to remain within ± 10% of the baseline level) have been reported to reduce the risk of postoperative organ dysfunction compared to that with standard management strategies (e.g., maintaining systolic blood pressure at no less than 90 mm Hg), especially in high-risk patients [11]. Therefore, when dealing with hypotension, it is essential to consider the patient’s baseline BP, risks of hypotension-related organ ischemia, and the nature of the procedure rather than maintaining a normal blood pressure value or defining hypotension using one threshold for different patient populations under different clinical situations [11–15]. Consequently, based on the above reasons and considering that the patient had chronic hypotension without clinical symptoms, we adopted an individualized blood pressure management strategy, maintaining the blood pressure at 70–90 mm Hg systolic and 55–65 mm Hg diastolic.

In parallel to these vital concerns, we should also closely consider the following aspects of anaesthesia in patients with SMS: First, we should pay attention to pre- and postoperative blood calcium levels to prevent hypercalcaemia and hypocalcaemia. Second, the patient should be moved gently during the perioperative period to prevent pathological fracture. Third, particular care should be taken to avoid volume overload in fluid management and use small doses of vasoactive drugs when necessary.

In summary, SMS is a rare but serious complication of dialysis-dependent ESRD patients with uncontrolled SHPT. In addition to the inherent pathophysiological characteristics of dialysis patients, patients often have altered skeletal and cardiovascular systems, which may lead to airway difficulties, heart valve disease, and hypertension or hypotension, posing a significant challenge to the anaesthesiologist. Therefore, the general condition should be optimized as much as possible preoperatively, with adequate assessment of the airway and cardiovascular system and appropriate refinement of preparation. The perioperative period should be closely monitored and carefully managed to maintain the stability of circulation and promote smooth rehabilitation after surgery.

## Data Availability

The datasets are available from the corresponding author on request.

## Abbreviations

SMS: Shrinking Man syndrome
ESRD: End-stage renal disease
SHPT: Secondary hyperparathyroidism
ASA: American Society of Anaesthesiologists
PTH: Parathyroid hormone
BNP: B-type natriuretic peptide
TTE: Transthoracic echocardiography
NYHA: New York Heart Association
HR: Heart rate
ABP: Arterial blood pressure
TEE: Transoesophageal echocardiography
